# Clinical effectiveness, cost-effectiveness and process evaluation of group schema therapy for eating disorders: study protocol for a multicenter randomized controlled trial

**DOI:** 10.1186/s40359-024-01624-w

**Published:** 2024-03-04

**Authors:** Suzanne H. W. Mares, Jeffrey Roelofs, Janôt Zinzen, Manouk Béatse, Hermien J. Elgersma, Ruben M. W. A. Drost, Silvia M. A. A. Evers, Annemarie A. van Elburg

**Affiliations:** 1 Department of Eating Disorders (Amarum), GGNet Mental Health, St. Annastraat 312c, Nijmegen, 6525 HG The Netherlands; 2 Clinical Psychological Science, Faculty of Psychology and Neuroscience, Experimental Psychopathology, Maastricht University, Maastricht, 6200 MD The Netherlands; 3 Department of Health Services Research, Care and Public Health Research Institute (CAPHRI), Faculty of Health, Medicine and Life Sciences (FHML), Maastricht University, Maastricht, the Netherlands; 4 Department of Clinical Psychology, Faculty of Social and Behavioural Sciences, Utrecht University, Utrecht, The Netherlands; 5 Department of Clinical Psychology & Experimental Psychopathology, University of Groningen, Groningen, the Netherlands; 6 Trimbos Institute, Centre for Economic evaluation and Machine Learning, National Institute of Mental Health and Addiction, Utrecht, The Netherlands; 7 Co-eur, Utrecht, The Netherlands

**Keywords:** (Atypical) Anorexia nervosa, (Atypical) Bulimia nervosa, Clinical effectiveness, Cognitive behavioral therapy, Cost-effectiveness, Cost-utility, Eating disorders, Economic evaluation, Group schema therapy, Randomized controlled trial

## Abstract

**Background:**

Eating disorders (EDs), such as (atypical) Anorexia (AN) and Bulimia Nervosa (BN), are difficult to treat, causing socioeconomic impediments. Although enhanced cognitive behavioral therapy (CBT-E) is widely considered clinically effective, it may not be the most beneficial treatment for (atypical) AN and BN patients who do not show a rapid response after the first 4 weeks (8 sessions) of a CBT-E treatment. Alternatively, group schema therapy (GST) may be a valuable treatment for this ED population. Even though GST for EDs has yielded promising preliminary findings, the current body of evidence requires expansion. On top of that, data on cost-effectiveness is lacking. In light of these gaps, we aim to describe a protocol to examine whether GST is more (1) clinically effective and (2) cost-effective than CBT-E for (atypical) AN and BN patients, who do not show a rapid response after the first 4 weeks of treatment. Additionally, we will conduct (3) process evaluations for both treatments.

**Methods:**

Using a multicenter RCT design, 232 Dutch (atypical) AN and BN patients with a CBT-E referral will be recruited from five treatment centers. Clinical effectiveness and cost-effectiveness will be measured before treatment, directly after treatment, at 6 and at 12 months follow-up. In order to rate process evaluation, patient experiences and the degree to which treatments are implemented according to protocol will be measured. In order to assess the quality of life and the achievement of personalized goals, interviews will be conducted at the end of treatment. Data will be analyzed, using a regression-based approach to mixed modelling, multivariate sensitivity analyses and coding trees for qualitative data. We hypothesize GST to be superior to CBT-E in terms of clinical effectiveness and cost-effectiveness for patients who do not show a rapid response to the first 4 weeks of a CBT-E treatment.

**Discussion:**

To our knowledge, this is the first study protocol describing a multicenter RCT to explore the three aforementioned objectives. Related risks in performing the study protocol have been outlined. The expected findings may serve as a guide for healthcare stakeholders to optimize ED care trajectories.

**Trial registration:**

clinicaltrials.gov (NCT05812950).

**Supplementary Information:**

The online version contains supplementary material available at 10.1186/s40359-024-01624-w.

## Background

Eating disorders (EDs) are associated with decreased social, psychological, and physical functioning in individuals [1] and elevated premature mortality rates [2]. On a healthcare system level, EDs contribute substantially to the mental health burden. Between 2000 and 2018, the weighted mean of point ED prevalence across continents had increased from 3.5 to 7.8% [3].

In a similar vein, the collective ED disease burden is reflected through major socioeconomic constraints. On average, ED patients generate more healthcare expenses and have lower employment rates than people without ED [4]. According to Van Hoeken and Hoek [5], over 3.3 million healthy life years are lost worldwide annually due to EDs. In addition to that, these authors showed that ED patients generated a 48% increase in healthcare costs, compared to the general population, while EDs with comorbid other psychiatric disorders were associated with a 48% decrease in yearly earnings in those employed.

In clinical practice, enhanced cognitive behavioral therapy (CBT-E) is used as treatment for all types of EDs, because of its transdiagnostic applicability and effectiveness [6]. Even though CBT-E is clinically effective for a certain group of ED patients, it is presumably not the most clinically optimized treatment for patients with (atypical) Anorexia Nervosa (AN) or Bulimia Nervosa (BN). More specifically, CBT-E is characterized by a substantial dropout rate: 20–54% in randomized controlled trials [6], especially in AN [7–9]. In general, only 30–50% of ED patients achieve clinical remission after CBT-E treatment [6, 10].

Although certain ED patients are greatly capable of challenging their own belief systems during treatment, they are generally unable to modify deeply held core beliefs [11]. In line with this observation, many ED cases reflect a complex intertwining between eating pathology and comorbid personality pathology [11–13], including clinical perfectionism [14]. Moreover, comorbid personality pathology increases the risk of attrition and is associated with greater psychopathology after treatment [11]. Albeit uncertain, not all (atypical) AN and BN patients may benefit sufficiently from CBT-E, perhaps due to a lack of emphasis on deeply rooted core beliefs.

In light of the findings above, schema therapy (ST) may be a clinically effective alternative that offers potential for addressing comorbid personality pathology. In short, ST provides an integrative approach that is fundamentally geared towards treating people with deeply entrenched self-identity difficulties and interpersonal problems, which are reflected through maladaptive schemas and schema modes [15]. Using these theoretical foundations, ST is either delivered individually or in a group setting (GST) in clinical practice. While individual ST [16, 17] and GST [18] were already found to be clinically effective and cost-effective for treating personality disorders, the rationale underlying their application for effectively treating EDs has also been documented [19]. The hypothesis is that EDs are characterized by overdeveloped coping modes that either avoid schema activation or manage affect when schemas are triggered. In general, the theoretical foundations regarding schemas and schema modes have already been applied to eating disorders in certain pilot studies, which were considered promising [20–22]. Contrary to individual ST, GST aims to establish a collective sense of awareness among ED patients regarding their shared emotional needs and applied schemas and coping mechanisms, building upon therapeutic alliance in the context of a supportive group environment. As a result, the GST approach may prevent drop-out rates among ED patients [23], which is a major hurdle in current CBT-E delivery formats.

New studies with adequate control groups are needed in order to expand the preliminary body of evidence on GST for eating disorders. This is important, since current CBT-E does not automatically address personality pathology in patients with persisting EDs, leaving many of them without adequate potential for recovery [8, 24]. In general, (atypical) AN and BN patients not showing an early treatment response after the first 4 weeks of a CBT-E treatment may be at an elevated risk of dropping out and achieving suboptimal clinical outcomes after a full CBT-E trajectory [25–27]. Therefore, the current study protocol will focus on ED patients who do not show a significant reduction in ED symptoms after the first 4 weeks (8 sessions) of CBT-E, which is the first of four CBT-E treatment phases [28].

Furthermore, to our current opinion studies on the cost-effectiveness of GST for ED patients are lacking. In accordance with a cost-effectiveness paradigm to facilitate value-based healthcare [29–31], cost evaluations of GST and CBT-E in proportion to their clinical outcomes are indispensable for the purpose of optimizing care trajectories for (atypical) AN and BN patients. Therefore, the current study protocol will examine the clinical effectiveness and cost-effectiveness of GST. This protocol aligns neatly with the Research Agenda in Dutch Mental Healthcare, which aims to improve two aspects: clinical value and affordability of mental healthcare in The Netherlands [32].

Implementation feasibility of new treatments in clinical practice is important. Ideally, new treatments should be easily implementable in clinical practice in order to avoid long term implementation barriers and to increase treatment successes [33]. Similarly, therapeutic alliance and patient experiences during treatment are generally considered predictors of clinical outcomes among ED patients [34]. In order to account for these predictive effects, process evaluations need to be conducted among participating ED patients and therapists during the implementation process of GST and CBT-E.

The current paper presents a comprehensive study protocol to examine the clinical effectiveness, cost-effectiveness and process evaluation of GST, compared to CBT-E, for patients with (atypical) AN or BN who do not show a significant reduction in ED symptoms after the first 4 weeks (8 sessions) of CBT-E treatment.

### Objectives

The current study protocol will test hypotheses and answer research questions from three distinct research areas:

#### Clinical effectiveness

We hypothesize GST to be more effective in terms of promoting treatment adherence and reducing ED severity, early maladaptive schema’s, dysfunctional schema modes and increasing quality of life, compared to CBT-E for (atypical) AN and BN patients not showing a rapid response after the first 8 CBT-E sessions.

#### Economic evaluation

We hypothesize GST to be more cost-effective from a societal perspective, compared to CBT-E, in terms of costs, effects and utilities.

#### Process evaluation

Within the process evaluation, research questions are (1) To what degree have the GST treatment and CBT-E treatment been delivered according to protocol? (2) What are reported reasons for protocol deviation in GST and CBT-E? and (3) What are the experiences of patients regarding GST and CBT-E?

### Methods/Design

#### Design

A multicenter randomized controlled superiority trial will be applied, in which GST is defined as the intervention treatment and continued CBT-E as the control treatment. Initially, patients assigned to a CBT-E treatment are eligible for screening. Before and after the first 8 CBT-E sessions, patients will be screened, using the Eating Disorder Examination Questionnaire (EDE-Q). Patients failing to reach a statistically defined cut-off score for reliable improvement on the EDE-Q (determined with the Reliable Change Index as described by Jacobson & Truax^35^), will be randomized to either GST or continued CBT-E as usual. These particular patients are called early non-responders to CBT-E. Conversely, patients reaching this cut-off score for reliable improvement on the EDE-Q will be excluded from further study participation prior to randomization and will continue their CBT-E treatment.

#### Randomization

**Fig. 1:**
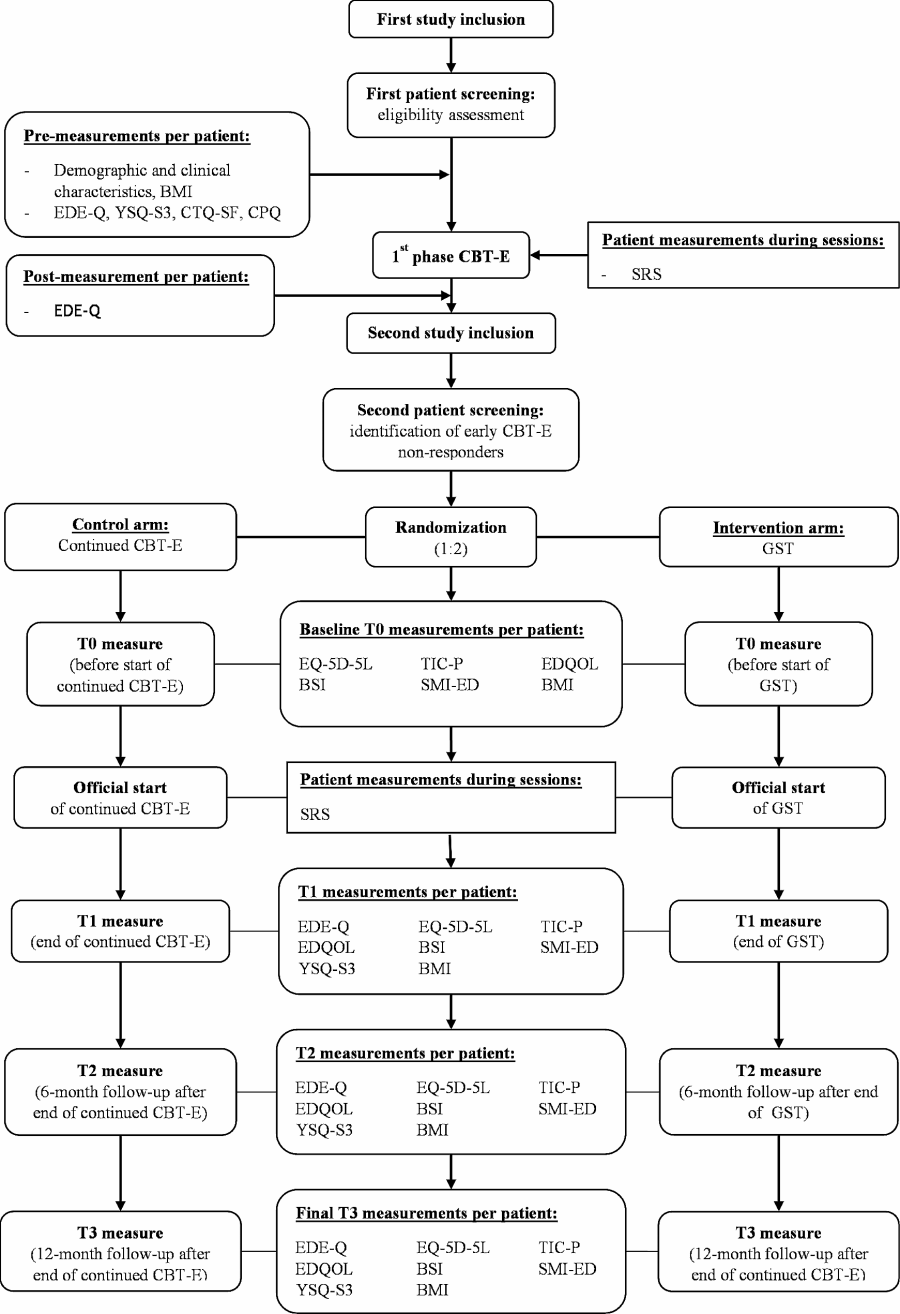
schematic representation of the study trial at each treatment location

Random allocation to the control group (continued CBT-E) or the intervention group (GST) will be conducted separately for each treatment location in a ratio of 1:2 by an automatized randomization software program after the completion of the post measurement. This ratio is due to the cluster effect in group-based therapy, which statistically requires more participants for the GST arm than for the continued CBT-E arm. Subsequent to randomization, participants in the GST arm and continued CBT-E arm will complete a baseline measurement (T0). After that, both treatments will start. Post-measurements (T1) will take place on the known end date of the treatment to which a participant is randomized. Follow-up measurements will take place 6 months (T2) and 12 months (T3) later, following initial completion of the entire treatment trajectory at T1. Several clinical measurement instruments are used, see Fig. 1. The current study protocol has been approved by the Medical Research Ethics Committee (MREC) Academic Hospital Maastricht/University Maastricht in The Netherlands, which was formally registered as: NL80491.068.22. The study is registered at ClinicalTrials.gov (registration number NCT05812950), and complies with the World Health Organization Trial Registration Data Set. Modifications to the protocol will be submitted as an amendment and will be examined by the MREC and will be adjusted in the trial registry.

### Study population

Patients are eligible for participation in the current trial, if they meet the following inclusion criteria: (1) an age of 16 years or older; (2) the ability to read and speak the Dutch language; (3) a DSM-5 classification of anorexia nervosa (AN), bulimia nervosa (BN), or other specified feeding and eating disorder (OSFED) that identifies as an atypical AN or BN variant with a low frequency or limited duration of binges / purges and (4) failure to show an early response after 8 sessions CBT-E. The Jacobson-Truax Reliable Change Index (RCI) approach will be used to assess whether the early CBT-E response is significant or not [35]. Exclusion criteria include: (5) the presence of an acute psychosis; (6) a clinically diagnosed autism spectrum disorder (ASD); (7) currently receiving schema therapy elsewhere and (8) an IQ below 80.

### Setting and recruitment of participants

Patients will be recruited from ED treatment centers in The Netherlands including GGnet Amarum (Warnsveld, Nijmegen), GGz Breburg (Tilburg, Breda), Co-eur (Hoensbroek, Maastricht, Roermond, Utrecht, Vught), GGZ Friesland (Leeuwarden), and Accare (Groningen). Patients meeting the eligibility criteria will receive an invitation from their therapist for study participation. After receiving written and oral information about the study from the researchers, each patient will decide individually on study participation. Once active consent has been provided, the patient will be included in the study trial, after which phase 1 CBT-E will start.

### Sample size

In order to reliably measure the quantitative primary outcomes following completion of the GST treatment and continued CBT-E treatment, a two-tailed alpha of 5%, a power of 90% and a medium effect size of d = 0.50 is assumed. This yields a sample size of 86 patients per treatment arm, thus *N* = 172 in total for a classical RCT. However, there is clustering in the GST arm due to its group format for treatment delivery. Therefore, an additional design effect is needed to correct the GST sample size for potential clustering. Assuming a group size of 8 participants and an intra-class correlation of 0.10, a design effect of 1.70 was computed. When multiplying this design effect with the original sample size of the GST arm (*N* = 86), the adjusted necessary sample size for the GST arm is equal to 1.70 *86 = 146 participants. Adding the initial number of participants in the continued CBT-E arm (*N* = 86), the total number of necessary study participants is equal to *N* = 232.

### Treatments

#### Intervention: Group Schema therapy (GST)

GST will consist of 26 weekly 90-minute group sessions, tailored to people with an ED. If necessary, GST can be supplemented with 8 individual sessions used for facilitating the GST process. The individual sessions can for example be used for imagery rescripting of adverse childhood experiences. Subsequent to randomization, patients may have to wait for at most three months before initiating their GST treatment in mental healthcare, as it is a half-open group, with an entry and exit moment every 13 weeks. During this waiting period, 5 individual treatment sessions will take place that focus on explaining the ST model, placing the ED symptoms and behaviors in the context of coping modes, and organizing these in a mode map conceptualization. This is an important foundation for the upcoming GST. The following sessions focus on recognizing and changing personal coping modes regarding eating disorder symptoms and underlying early maladaptive schemas. These sessions are also aimed at developing and strengthening the healthy adult mode. In doing so, GST combines interpersonal, experiential, cognitive and behavioral elements in a unified ST approach. On top of that, a psycho-education webinar will be organized for relatives.

**Theoretical framework for GST: a group format of ST.** In short, ST provides an integrative approach that is fundamentally geared towards treating people with deeply entrenched self-identity difficulties and interpersonal problems, which are reflected through maladaptive schemas and schema modes [15]. In clinical practice, ST was deemed effective for treating personality disorders [16], including ST in a group setting [18]. On the one hand, maladaptive schemas refer to a pervasive pattern of beliefs regarding oneself and one’s relationship with others consisting of one’s memories, emotions, cognitions and bodily sensations. On the other hand, schema modes are characterized by one’s moment-to-moment thoughts, feelings and behaviors when maladaptive schemas interact with coping mechanisms [23].

In terms of treatment delivery, ST emphasizes experiences, while CBT-E mainly emphasizes cognitions and behavior. In GST, the connection between participants in the group format is considered an amplifier for the effects of ST. On top of that, GST ensures a healthy therapeutic relationship to provide corrective experiences in favor of the patient’s needs during treatment [23].

#### Control treatment: individual enhanced cognitive behavioral therapy for eating disorders (CBT-E)

In general, CBT-E is a transdiagnostic ED treatment, meaning that it is widely used for most diagnosed EDs in mental healthcare. In addition to that, it is also considered the current standard of care for most EDs. An entire CBT-E trajectory consists of 20–40 individual sessions, each of which will last 60 min at most. In the current protocol, the first 8 CBT-E sessions for a patient will take place before randomization (phase 1 CBT-E). After being randomized to the continued CBT-E arm, the remaining 12–32 individual sessions will take place.

From a theoretical perspective, CBT-E is based on a so-called transdiagnostic CBT model consisting of four phases. In phase one, patients formulate a personal goal, in which they learn to monitor and establish regular eating behaviors in order to address their eating disorder. In doing so, patients are offered psycho-education on factors that are maintaining their eating disorder. In phase two, consisting of one session, a more specific treatment plan is developed, based on an evaluation of the first phase. During phase three, the identified main mechanisms that are hypothesized to maintain the patient’s ED are addressed. These are (1) an excessive evaluation of body image, (2) dietary restraint, and (3) events, mood and eating. Subsequently, a relapse plan is developed in order to maintain the newly established eating behaviors when faced with these challenging mechanisms. Lastly, phase four aims to evaluate and sustain the total progress that has been achieved during the prior phases.

#### Discontinuation/modification of treatment

In case of acute crisis, the crisis procedures of the treatment centers will be followed. Any additional treatment will be recorded by the therapists and included in the analyses. Patients will only be withdrawn from the study at their request. All (serious) adverse events reported either by the patient, the clinicians, or researchers will be recorded.

### Data collection

Collection of all outcome data will take place on multiple occasions during the trial. Prior to randomization, the EDE-Q will be measured on two different points in time: during the pre-measurement and post-measurement of phase 1 CBT-E. Additional clinical instruments will be used during the pre-measurement of phase 1 CBT-E to measure demographic and clinical characteristics, childhood trauma, perfectionism, and early maladaptive schemas at baseline.

In order to practically operationalize data collection, a digital data handling system will be used. In short, this system is aimed at facilitating informed consent, recruiting and guiding each new research participant automatically to all online measurements in accordance with privacy regulations in Dutch mental healthcare, based on each participant’s individual care trajectory and timeline during the trial. Using an iterative approach with test data to eliminate dysfunctional steps in this automated process, a sophisticated data collection system will be established, presenting all questionnaires in an organized manner with encouraging cartoon pictures. Each measurement will be completed either through hand-based devices (e.g. a smart-phone) or on a computer. From a design perspective, the possibility of using different devices and showing encouraging cartoon visuals may motivate participants to complete online surveys [36]. Data is collected with a pseudonym for each patient. A list of pseudonyms and identifying information of patients is securely stored at the mental healthcare centers and only accessible for the research assistant and coordinator of this center and the authorized researchers. The data is stored on a secure storage server of Maastricht University, accessible only to authorized researchers. The project leaders will be responsible for data monitoring. The study will not be audited. The results of the study will be disseminated in scientific and clinical journals and presentations at (inter)national scientific and clinical conferences.

Subsequent to randomization, participants will complete measures before continued CBT-E or the start of GST (T0), immediately after the confirmed end date of treatment (T1), and 6 months (T2) and 12 months (T3) following completion of treatment. In addition to measuring perceived ED schemas, data on quality of life and healthcare service use will also be gathered on these occasions. Data on process evaluations, as assessed by session rating scales and patient registration files, will be collected during each treatment session of phase 1 CBT-E, continued CBT-E, and GST. After treatment (T1), a representative sample of ED patients from both arms will be recruited to participate in a qualitative interview to gain deeper insights into their treatment experiences. Due to the nature of the interventions, blinding of therapists, interviewers, and patients is not possible. Below, all measures are stratified for clinical effectiveness, economic evaluation and process evaluation.

#### Clinical effectiveness

A distinction is made between primary and secondary outcome data in order to assess clinical effectiveness.

I. Primary outcome.

ED pathology is the primary outcome measure, which will be assessed using the Eating Disorder Examination-Questionnaire [37]. The EDE-Q is a valid and reliable instrument [38], which is sensible to change [39].

II. Secondary outcomes.

Early maladaptive schemas will be measured, using the Young Schema Questionnaire Short Form version 3 (YSQ-S3). It is considered a valid and reliable instrument for the purpose of assessing maladaptive schemas in clinical and research settings [40, 41].

The Schema Mode Inventory for Eating Disorders (SMI-ED) will be applied to identify specific schema modes that are relevant in ED patients. In short, it measures maladaptive child modes, maladaptive internalized modes, maladaptive coping modes and healthy factors, and has adequate validity and high reliability [42].

To rate general psychological and physical symptoms the Brief Symptom Inventory (BSI) will be used, which is highly validated and very sensible to detecting change [43].

Quality of life in ED patients will be assessed using the Eating Disorder Quality of Life (EDQOL) questionnaire. The EDQOL focuses on distinct life domains that are relevantly impacted by eating disorders, and has shown good validity, reliability and sensitivity [44].

Generic quality of life will be measured using the well validated EQ-5D-5 L [45, 46]. This instrument aims to measure quality of life objectively, using five questions regarding quality of life in five distinct domains: mobility, self-care, usual activities, pain / discomfort, and anxiety / depression. Additionally, a 100 mm VAS scale is used to assess a patient’s perceived quality of life [47].

Clinical perfectionism will be assessed using the Clinical Perfectionism Questionnaire (CPQ). In short, this 12-item questionnaire measures the extent to which an individual strives towards extremely high personal standards in the face of adverse consequences. The CPQ is deemed a reliable measure in ED samples [48, 49].

Since childhood trauma may affect ED treatment outcomes, the validated Childhood Trauma Questionnaire-Short Form (CTQ-SF) will be included. It consists of 28 questions measuring physical abuse, emotional abuse, sexual abuse, physical neglect and emotional neglect during childhood [50].

Demographic and clinical characteristics will be identified at baseline, using a tailored list of questions on current treatment expectations, gender, socioeconomic status (labor and education), prior ED treatments and living situation.

Since BMI can be an important indicator of somatic improvement in EDs, it will be measured repeatedly during the study trial by asking the participants about their height and weight.

#### Economic evaluations

Societal costs will be determined using the Treatment Inventory of Costs in Patients with psychiatric disorders questionnaire (TIC-P). This validated instrument assesses costs by measuring consumed healthcare resources and productivity losses [51]. Lastly, the economic evaluation study will be performed in line with the Dutch Guidelines of the National Health Care Institute [52].

During the entire data collection phase, the TIC-P and the aforementioned clinical instruments will be mostly measured on the same time points (T0-T3). See Table 1.

**Table 1:** all questionnaires for each measurement point in time

Instruments	Time points^a^					
	Pre-measure	Post-measure	T0	T1	T2	T3
Demographic and clinical characteristics	x					
EDE-Q	x	x		x	x	x
TIC-P			x	x	x	x
EQ-5D-5 L			x	x	x	x
EDQOL			x	x	x	x
SMI-ED			x	x	x	x
YSQ-S3	x			x	x	x
CTQ-SF	x					
BSI			x	x	x	x
CPQ	x					
BMI	x		x	x	x	x

^a^ Pre-measure: measurement before the initial session of CBT-E, post-measure: measurement after 8 sessions of CBT-E, T0: baseline measurement, T1: measurement following the end of treatment, T2: measurement at 6-month follow-up, T3: measurement at 12-month follow-up

#### Process evaluation

In order to measure the extent to which GST is a feasible alternative to CBT-E in clinical practice for patients and therapists, process evaluations will be conducted. More specifically, treatment fidelity (treatment quality), dose delivered (completeness), dose received (exposure and satisfaction), and reach (participation rate) will be assessed in both the GST arm and the continued CBT-E arm.

I. In order to assess dose received, i.e. treatment satisfaction and overall therapeutic alliance, patients will complete a session rating scale (SRS) during each treatment session. The SRS consists of 4 questions regarding key dimensions of effective therapeutic relationships [53].

II. To assess dose delivered and reach, therapists will complete patient registration files after each session. In these files, therapists can describe patient attendance and the extent to which treatment was delivered according to protocol, including potential deviations and additional therapies or medications. These files are expected to assess the true implementation characteristics of the treatment protocol in Dutch clinical practice.

III. Recording of therapy sessions will take place regularly in order to rate overall compliance to the treatment protocol, quality of treatment delivery and the number of patients that were able to participate (fidelity, dose delivered and reach).

IV. After the end of treatment, qualitative interviews will be conducted among certain patients from the GST arm and the continued CBT-E arm, which will take approximately 30 min. Using existing guidelines for qualitative data collection and analysis [54], these interviews are aimed at generating original findings regarding patient experiences that are not identifiable through quantitative approaches. Building upon the SRS, these interviews may identify profound data regarding treatment satisfaction and therapeutic alliance.

### Data analysis

Below, a general plan has been developed to analyze data on clinical effectiveness, economic evaluation and process evaluation. In order to conduct quantitative analyses, SPSS and R will be used. Nvivo, which is a coding software tool, will be used to convert and analyze qualitative data from the patient interviews. Data analyses will be conducted according to the intention-to-treat principle, meaning that analysis of all trial results is entirely based on the initial treatment arms to which all patients were assigned after randomization, regardless of whether they actually received that treatment or not. Data-analysts will be blind for condition. Covariates, including sex, age, prior treatment and gender, will be accounted for in all (baseline) analyses to ensure the comparability of both treatment arms after proper randomization. Lastly, a two-sided significance level of 0.05 will be applied to test statistical significance.

#### Clinical effectiveness

Using t-tests and Chi-square tests, differences between the GST arm and CBT-E arm regarding demographic and clinical characteristics will be described. Dropout analysis among patients will be corrected for relevant background characteristics in order to examine the representativeness of our study population during the trial. In case dropout is selective, follow-up analyses will take this phenomenon into account. Relevant background characteristics may include gender, age and diagnosis. After treatment, change scores will be determined in multilevel analyses for both treatment arms between T0 and T1, T1 and T2, and T2 and T3. Suitable model assumptions will be used in order to account for the potential measurement correlations between these time points and nested data structures. Likewise, the significance of change scores between both arms will be determined at each time point, using a regression based mixed models approach. In case of significant effects, suitable post hoc analyses will be conducted. If possible, clinical findings will be at least stratified for gender and age in both treatment arms. In order to examine the robustness of clinical findings, sensitivity analyses will be performed, including the analysis of missing data. Based on the intention-to-treat principle, missing value analysis in SPSS will be used in order to estimate expected values of missing outcomes among participants remaining in the analysis. Based on the quality, completeness and statistical distributions of the data obtained, suitable missing data handling procedures will be applied.

#### Economic evaluation

**Base-case analysis.** Using a micro-costing and bottom-up approach, total costs will be approximated from a societal perspective in the GST arm and the continued CBT-E arm, in line with recent best practices [52]. Essentially, each element of service use will be multiplied by an appropriate unit cost in euros. If necessary, unit costs will be updated, using the 2023 consumer price index [55]. Relevant unit costs for each type of service are outlined in contemporary Dutch costing manuals for healthcare services [56, 57]. Intervention costs, healthcare costs, patient and family costs and productivity losses for unpaid work, paid work and education will be estimated. Productivity losses will be quantified, using a friction cost approach that is confined to the time period required to replace one sick employee. In case of major uncertainty surrounding the abovementioned analyses, cost estimates will be founded on the most conservative assumptions.

Primarily, economic evaluations aim to compare the additional costs and outcomes of GST to those of CBT-E. In the economic evaluation of the current protocol, collected data will be used to conduct a cost-effectiveness analysis (CEA) and a cost-utility analysis (CUA). On the one hand, a CEA expresses all clinical treatment effects of GST and CBT-E relative to their economic costs, using the incremental cost-effectiveness ratio (ICER). On the other hand, a CUA aims to compare all gains in quality adjusted life years (QALYs) of GST and CBT-E to their respective economic costs, using the incremental cost-utility ratio (ICUR).

In short, the ICER is aimed at dividing the difference in treatment effects of GST and CBT-E by the difference in total costs of both treatments. For instance, the total difference in clinical EDE-Q gains of both GST and CBT-E can be weighed against the cost differences of both treatments. Using a similar incremental cost-utility ratio (ICUR), the difference in QALY gains of both treatments is divided by their respective cost differences. QALYs are computed based on the measured EQ-5D-5 L data, using converted EuroQol utility scores [58].

In order to verify the robustness of the obtained ICER and ICUR, non-parametric bootstrapping techniques will be conducted. These bootstrap replications are characterized by random sampling based on individual input data, yielding information about the magnitude and distribution of most pairwise cost-effect estimates. In order to facilitate this objective, the bootstrap replications will yield the 95% confidence intervals for the obtained ICER and ICUR within a cost-effectiveness plane, surrounding the 2.5th and 97,5th percentiles [29]. While the horizontal axis of a cost-effectiveness plane depicts the difference in effectiveness between two treatments, the vertical axis represents the cost difference between two treatments [59].

Subsequently, a cost-effectiveness acceptability curve will be plotted in order to determine the maximum amount of money that society is willing to pay for one unit of clinical improvement or one unit of QALY gains. This maximum amount of money is also called the willingness-to-pay (WTP) ceiling ratio [29]. In the Dutch healthcare system, ceiling ratios of € 20,000, € 50,000 and € 80,000 per QALY exist, depending the disease burden that will be determined based on the study population [60]. If the computed ICER or ICUR for a certain treatment is likely to fall below these thresholds, that treatment is usually considered cost-effective. In order to determine whether GST is cost-effective over CBT-E in the Netherlands, the cost-effectiveness acceptability curve will display the probability that the ICER and ICUR of GST fall below a range of existing ceiling ratios. This computed probability is based on the obtained bootstrap replications for the ICERs and ICURs of GST. Lastly, multivariate sensitivity analyses will be conducted to examine whether different assumptions regarding cost prices and volumes may affect the obtained ICERs and ICURs.

**Budget impact analysis.** Subsequent to these base-case analyses, a budget impact analysis (BIA) will be conducted according to existing quality guidelines [61, 62]. In short, this BIA aims to inform Dutch healthcare stakeholders on the foreseeable financial consequences of implementing GST on a regional or national level. Importantly, the BIA will be tailored to the relevant cost categories for separate stakeholders. Appropriate assumptions regarding data distribution of cost components will be developed in order to objectively quantify the budget impact of GST for a variety of stakeholders over a 3-year time horizon. In order to do so, the BIA will target at least four parameters: (1) the national prevalence of the eligible GST intervention population in The Netherlands, (2) the expected GST implementation rate in clinical practice, (3) average GST trajectory costs, and (4) average CBT-E trajectory costs. All four parameters mentioned above will be subjected to univariate and multivariate sensitivity analyses in order to estimate the robustness of the national GST budget impact in different scenarios. Lastly, the BIA will be conducted from a societal, healthcare and commercial payer perspective.

#### Process evaluation

Data on process evaluation will be analyzed, using qualitative and quantitative methods. Data from the qualitative interviews will be used to explore and distill relevant themes regarding treatment experiences among patients during the trial.

In principle, we aim to record 10% of administered sessions with audio devices in order to assess whether treatment sessions have been implemented according to protocol. Furthermore, quantitative data from items on the session rating scales (SRS) will be analyzed, using descriptive statistics in SPSS. For example, descriptive statistics per SRS item in both treatment arms may include: medians, means and frequencies. Using t-test analysis, differences in SRS outcomes between both treatment arms will be examined quantitatively in order to determine whether GST is more feasible than CBT-E for patients.

## Discussion

By illuminating the clinical effectiveness of GST for treating EDs, including the interconnections between ED severity and schemas, our study findings may expand the existing body of evidence on the clinical potential of GST for treating (atypical) AN and BN patients not showing a rapid response after the first phase (8 sessions) of CBT-E. Due to unsatisfactory CBT-E treatment rates and the absence of a clinically dominant alternative in current practice for a substantial group of patients [6, 10], we are morally obliged to aim at developing effective treatment alternatives for this group. Taking into account ED healthcare and societal costs [4], the current study protocol will also examine whether GST is a cost-effective and feasible alternative to CBT-E in a specific subgroup from a health economic perspective.

### Study strengths

To our knowledge, this is the first study protocol initiating a multicenter RCT trial in clinical practice to examine the clinical effectiveness, cost-effectiveness and feasibility of GST as an alternative to CBT-E for eating disorder on a global scale. The multicenter RCT research design, enabling collaborations between research and clinical practice and people with lived experience, may greatly facilitate the organizing process of the current trial and may expand the existing body of evidence on GST in real clinical settings. As a result, our study may generate relevant impact in Dutch mental healthcare beyond academia. Using a declaration of intent, all institutions have declared their willingness to participate in the current trial.

Second, an extensive data collection procedure has been developed, enabling broad analyses of clinical effectiveness and cost-effectiveness. In addition to that, all clinical measurement instruments used are highly validated. Primarily, the EDE-Q [37], YSQ-S3 [40, 41], SMI-ED [42], EDQOL [44] and EQ-5D-5 L [45] have been applied extensively in the field of psychotherapy research.

Third, the tested data handling system mentioned earlier is expected to greatly facilitate and automate patient recruitment, consent and data collection, which is tailored to each patient’s individual timeline during the trial. Using these tested and automated processes, data collection is expected to unfold in a standardized manner, which may reduce the risk of incomplete information, major deviations and related errors in the collection process. If necessary, this system can always be adapted, based on emerging observations during data collection.

Last, the TIC-P questionnaire was tailored to our population of interest through a careful expert review. In a similar vein, the layout and length of the TIC-P has varied over time in previous research [63]. Health services research on initiatives for measuring worker wellbeing has illustrated the importance of tailored questionnaires in order to highlight aspects that are relevant to participants [64]. In light of these previous findings, a tailored and shorter TIC-P version is expected to capture the most prevalent cost categories among ED patients, making it more suitable for routine outcome monitoring in the current project. This may reduce the risk of incomplete data resulting from ED patients failing to align with a questionnaire that is not fully relevant to them.

### Operational challenges

Although we are confident about the general applicability and resilience of the current study protocol, there are certain operational challenges that require our consideration.

First, ED patients are at risk of comorbid physical and mental health complications [65]. Even though severely vulnerable and ill-protected ED patients will be excluded during the screening phase, we cannot rule out the possibility of ED patients terminating their research participation due to health complications in a subsequent research phase. As a result, there is an inherent risk of incomplete data, which we aim to address by conducting all statistical analyses according to the intention-to-treat principles. Additionally, information leaflets are tailored to the research treatment phases that apply to participating patients, supporting their treatment adherence.

Second, participating ED treatment centers could face implementation challenges due to several barriers [66], including financial constraints and management challenges on the organizational level. In order to address these issues, regular communication strategies and facilitation of the implementation processes are key. In light of this objective, we aim to organize regular meetings and we will establish consistent communication lines, both online and on-site. Newsletters are prepared regularly in order to convey important updates and to optimize the overall sense of community among our participating partners.

Third, completion of all measurements may require substantial efforts from participants. Given the number of relevant questionnaires being administered, a full measurement may take up to two hours to complete. Although encouraging visuals will be implemented digitally to facilitate completion of each measurement over an extended time span with regular breaks, completing all measurements in time may still be challenging for participants. If possible, completion of measurements will be supported on-site at the institutions.

Fourth, it may be difficult to identify which components or sessions of GST are most (cost-)effective, in case overall (cost-)effectiveness of GST is shown. In reality, different factors contribute to treatment success that may be indirectly related to GST and CBT-E delivery, including therapeutic alliance. In spite of this particular limitation, the extensive data collection procedures, including a process evaluation and qualitative interviews, are expected to generate valuable and broad insights regarding the (cost-)effectiveness of GST.

Last, all participating therapists are qualified in the field of GST: they are experienced in leading a patient group and they have completed a basic GST course. However, not all participating therapists will be equally familiar with GST principles from the start. In order to ensure consistent treatment delivery among patients, an overarching supervision plan for all participating treatment centers is developed and tailored to their existing organizational structures. Being an authority in the field of ST for eating disorders [23], Susan Simpson’s e-learnings will be provided to all participating therapists. These educational courses will be the foundation for discussion points during the overarching supervision meetings.

### Implications for healthcare policy and clinical practice

In the current project, we aspire to pave the way towards clinically meaningful and affordable care trajectories in daily practice for (atypical) AN and BN patients not showing a rapid response after the first phase (8 sessions) of CBT-E. In light of this objective, we aim to inform clinical practice, Dutch healthcare policymakers and insurance companies about the extent to which GST provides solid clinical value for money, as an alternative to the existing CBT-E. Given the substantial ED disease burden for both individuals and society as a whole, future research initiatives examining the clinical effectiveness and cost-effectiveness of GST are highly warranted.

### Electronic supplementary material

Below is the link to the electronic supplementary material.

Supplementary Material 1

## Data Availability

No datasets were generated or analysed during the current study.

## Abbreviations

AN: Anorexia nervosa
BN: Bulimia nervosa
CBT-E: Cognitive behavioral therapy - enhanced
ED: Eating disorder
GST: Group schema therapy
ICER: Incremental cost-effectiveness ratio
ICUR: Incremental cost-utility ratio
ST: Schema therapy
WTP: Willingness to pay
